# Distinguishing between Decaffeinated and Regular Coffee by HS-SPME-GC×GC-TOFMS, Chemometrics, and Machine Learning

**DOI:** 10.3390/molecules27061806

**Published:** 2022-03-10

**Authors:** Yun Zou, Meriem Gaida, Flavio A. Franchina, Pierre-Hugues Stefanuto, Jean-François Focant

**Affiliations:** 1 Organic and Biological Analytical Chemistry Group, MolSys Research Unit, University of Liège, 4000 Liège, Belgium; meriem.gaida@uliege.be (M.G.); phstefanuto@uliege.be (P.-H.S.); jf.focant@uliege.be (J.-F.F.); 2 Department of Chemical, Pharmaceutical and Agricultural Sciences, University of Ferrara, 44121 Ferrara, Italy; flavioantonio.franchina@unife.it

**Keywords:** coffee, decaffeination, aroma profile, solid-phase microexaction, two-dimensional gas chromatography, time-of-flight mass spectrometry, PCA, *t*-test, PLS-DA, random forest

## Abstract

Coffee, one of the most popular beverages in the world, attracts consumers by its rich aroma and the stimulating effect of caffeine. Increasing consumers prefer decaffeinated coffee to regular coffee due to health concerns. There are some main decaffeination methods commonly used by commercial coffee producers for decades. However, a certain amount of the aroma precursors can be removed together with caffeine, which could cause a thin taste of decaffeinated coffee. To understand the difference between regular and decaffeinated coffee from the volatile composition point of view, headspace solid-phase microextraction two-dimensional gas chromatography time-of-flight mass spectrometry (HS-SPME-GC×GC-TOFMS) was employed to examine the headspace volatiles of eight pairs of regular and decaffeinated coffees in this study. Using the key aroma-related volatiles, decaffeinated coffee was significantly separated from regular coffee by principal component analysis (PCA). Using feature-selection tools (univariate analysis: *t*-test and multivariate analysis: partial least squares-discriminant analysis (PLS-DA)), a group of pyrazines was observed to be significantly different between regular coffee and decaffeinated coffee. Pyrazines were more enriched in the regular coffee, which was due to the reduction of sucrose during the decaffeination process. The reduction of pyrazines led to a lack of nutty, roasted, chocolate, earthy, and musty aroma in the decaffeinated coffee. For the non-targeted analysis, the random forest (RF) classification algorithm was used to select the most important features that could enable a distinct classification between the two coffee types. In total, 20 discriminatory features were identified. The results suggested that pyrazine-derived compounds were a strong marker for the regular coffee group whereas furan-derived compounds were a strong marker for the decaffeinated coffee samples.

## 1. Introduction

Coffee is one of the most popular beverages in the world [1]. It accounted for 65% of the hot drinks market worldwide in 2019 [2]. Caffeine is a key compound in both green and roasted coffee beans as it contributes 1.1 wt% for Arabica and 2.2 wt% for Robusta coffee beans [1]. Caffeine can block adenosine, thereby on the one hand increasing alertness and arousal, but on the other hand reducing sleep quality [3]. Caffeine also has some adverse effects on gastrointestinal disturbance, palpitation and increasing blood pressure [4]. Some consumers who are sensitive to the stimulating effect of caffeine prefer avoiding caffeine and choose decaffeinated coffee instead. The consumption of decaffeinated coffee was around 5% and 20% in Germany and the US, respectively, between 2009 and 2014 [1]. The global decaffeinated coffee market size was USD 1.65 billion in 2019 and is expected to grow in the near future [5]. 

The maximum caffeine content in decaffeinated coffee products is different around the world. Most European countries, the US, and Canada restrict a maximum of 0.1 wt% caffeine in green and roasted coffee [1]. Since 1908, the first successful decaffeination process was patented, and the classical sequence of pre-wetting the green coffee beans with water, extracting caffeine, and drying the beans has been applied on an industrial scale. There are three caffeine extraction approaches, using organic solvents, water, and liquid or supercritical carbon dioxide (LiCO_2_ or scCO_2_). Commonly used organic solvents are methylene chloride (DCM) and ethyl acetate (EA) [1]. Decaffeination by supercritical CO_2_ has become a trend because of its advantages, such as it being harmless, non-flammable, and having outstanding selectivity.

Removing the caffeine from coffee bean cells can cause unwanted side effects such as mass loss and solvent residues. The most direct effect to decaffeinated coffee drinkers is the relatively plain and thin taste [6]. This is due to the fact that the roasting process, which produces aroma compounds, is conducted after the decaffeination process [1]. Some aroma precursors may be co-extracted with caffeine during decaffeination. Therefore, the aroma cannot be vastly formed during roasting. A previous study reported a lower content of alkylpyrazines in decaffeinated coffee compared to regular coffee, which may be a result of the decaffeination process [7]. The earthy aroma significantly contributed by the presence of pyrazines was reduced as well [1,8]. Few papers have been published regarding the influence of the decaffeination process on coffee flavor. However, the flavor change of decaffeinated tea has been well-investigated in both green and black tea. It has been observed that the more caffeine was removed, the more volatile compounds were reduced. Some aroma-active compounds even disappeared after the decaffeination process [4,9,10]. 

Conventional chemometric methods such as principal component analysis (PCA), hierarchical cluster analysis (HCA) and partial least squares-discriminant analysis (PLS-DA) have been extensively used in food analytical chemistry [11] in general, and in particular in the study of the aroma profile of coffee [12,13,14]. However, in recent years, multiple works have pointed out the perks of using machine-learning (ML) algorithms such as support vector machine (SVM), classification and regression tree (CART) and random forest (RF) to assess food quality and authenticity. They have also reported their advantages compared to conventional statistical tools [15]. These ML algorithms have yielded promising results when used in detecting food fraud, i.e., substituting original ingredients with cheaper ingredients [16], investigating food origin [17], and detecting food additives [18]. Although, CART and RF have provided valuable results in the area of food chemistry, they remain more frequently used in “omics”-related fields. SVM is the most commonly used ML algorithm in food analysis [15].

This study aimed to understand the influence of the decaffeination process on the volatile compound composition using both targeted and non-targeted analysis. Using state-of-the art headspace solid-phase microextraction two-dimensional gas chromatography time-of-flight mass spectrometry (HS-SPME-GC×GC-TOFMS) enabled us to obtain comprehensive information on volatile compound profiles by analyzing the headspace of ground coffee. The difference of aroma-related volatile composition between decaffeinated and regular coffee was revealed by targeted analysis and chemometric tools. A non-targeted analysis using the random forest machine-learning algorithm was used to investigate the candidate chemical features that best divide the two coffee types (regular and decaffeinated). 

## 2. Results and Discussion

### 2.1. Targeted Analysis of Aroma-Related Volatile Compounds

#### 2.1.1. Aroma-Related Volatiles

Coffee is a complex matrix. During the roasting process, the precursors in the green beans are transformed into compounds that determine the aroma and taste of coffee. Major chemical reactions are the Maillard reaction, Strecker degradation, caramelization, degradation of chlorogenic acids and lipid oxidation [19]. Although there are hundreds of volatile compounds identified in roasted coffee, only dozens of them significantly contribute to the aroma profile [19]. Summarizing the Arabica coffee marker list from previous research [20], the volatile compound list in roasted coffee and the main odor-active compound list from two review articles [8,21], then searching in the coffee samples measured in this study, a total of 52 aroma-related volatile compounds were finally selected for target analysis (Table 1). This aroma-related volatile list included 4 acids, 3 alcohols, 6 aldehydes, 6 ketones, 9 furans, 1 lactone, 3 phenols, 13 pyrazines, 2 pyridines, 3 pyrroles, and 2 sulfur-containing compounds. Three pairs of coffee (R-R vs. D-R, R-F vs. D-F, R-VO vs. D-VO, Table 2) were used to investigate the difference in aroma-related volatiles between regular and decaffeinated coffee. These three pairs were selected because in each pair, the origin of the coffee beans, the species of the coffee beans (*Coffea arabica* L. (Arabica) or *Coffea canephora Pierre* (Robusta)), and the roasting process were identical. The only difference was the presence or the absence of the decaffeination process.

#### 2.1.2. Chemical Families of Aroma-Related Volatiles

**Figure 1:**
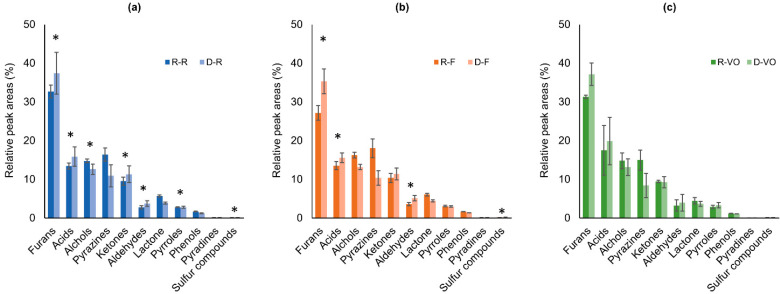
The comparison of targeted aroma-related volatiles between regular and decaffeinated coffee ((**a**). R-R vs. D-R, (**b**). R-F vs. D-F, (**c**). R-VO vs. D-VO) according to the chemical family relative peak areas. In the abbreviations of the coffee samples names, the initial letters R and D indicate the coffee type: regular and decaffeinated. The second letters R, F, and VO indicate the label of the coffee capsule. The asterisk signs (*) displayed above certain chemical families indicate a significant statistical difference (*p* < 0.05, *t*-test) between the regular and the decaffeinated coffee.

As shown in Figure 1, some chemical families such as alcohols, pyrazines, lactones, pyrroles, phenols, pyridines, and sulfur-containing compounds showed higher peak areas in the regular coffee than in the decaffeinated coffee. The reduced formation of pyrazines is previously observed in DCM decaffeinated coffee [1]. The relatively lower content of alkylpyrazines in decaffeinated ground coffee was also reported [7]. Pyrazines are the principle pyrolysis products of sucrose during the roasting process [14]. Furthermore, a significant amount of sucrose is removed unintentionally together with caffeine during the decaffeination process [22,23]. In fact, 60% and 20% losses in sucrose content were observed in the green beans of *Coffea arabica* L. (Arabica) and Coffea canephora Pierre (Robusta), respectively, after decaffeination by DCM [22,23]. Therefore, this explains why pyrazines were less concentrated in the decaffeinated coffees than in the regular coffees. During the roasting process, the pyrolysis of sucrose can also produce pyrroles [22]. Therefore, the number of pyrroles was relatively lower in the decaffeinated group. Besides the sucrose, chlorogenic acids are also partially removed during decaffeination (16% loss for Arabica and 11% loss for Robusta) [23]. Chlorogenic acids are the precursors of phenolic derivatives and lactones [21,22]. The content of phenolic compounds in the roasted ground coffee is related to the content of chlorogenic acids in the green beans [8]. Thus, this explains the lower content of phenols and lactones in the decaffeinated group. The decaffeination process extracts little amounts of proteins, lipids, and trigonelline as well. The roasting process degrades trigonelline into pyridines and pyrroles. During the Maillard reaction, the degradation of trigonelline, protein, and sucrose produces pyridines, pyrroles, and pyrazines [21]. Lipids are the precursors of alcohols. The decrease in lipids in decaffeinated green beans results, therefore, in the lower content of alcohols in roasted decaffeinated beans.

In contrast, furans, acids, ketones and aldehydes showed higher peak areas in the decaffeinated coffees than the regular coffees (Figure 1). The coffee bean color turns to light brownish after the decaffeination process [1]. In a roasted coffee production site, the completion of the roasting process is partially decided by the color of the beans. Therefore, to achieve the same final color, the decaffeinated beans may need shorter time than the regular green beans, which means the decaffeinated beans may be less roasted. Generally, less roasted coffee presents more acidity [19]. This may explain the higher acid content in the decaffeinated coffees. The amount of some compounds such as diketones decrease with excessive roasting because they are formed in the early stages of roasting [19]. Therefore, less roasting time of decaffeinated coffee cause higher ketone content. Aldehydes that impact flavor quality are produced from two pathways. The Strecker aldehydes, such as 2-methylbutanal and 3-methylbutanal, are the products of the Strecker degradation [24]. The precursors involved in Strecker degradation are amino acids and diketones derived from the Maillard reaction. The other pathway is from lipid oxidation of unsaturated fatty acids [19]. In the Strecker degradation process, more Strecker aldehydes can be produced from more diketones. This may partially illustrate the higher aldehydes and ketones content observed in decaffeinated coffees. Furans are Maillard reaction products [25]. The formation of furans involves thermal lipid oxidation, thiamine and nucleotide degradation, and thermal degradation of D-glucose and sugar polymers [8]. The reasons for higher furan content in decaffeinated coffee are unclear to the knowledge of the authors.

#### 2.1.3. Differentiation of Regular and Decaffeinated Coffee by Aroma-Related Volatiles

**Figure 2:**
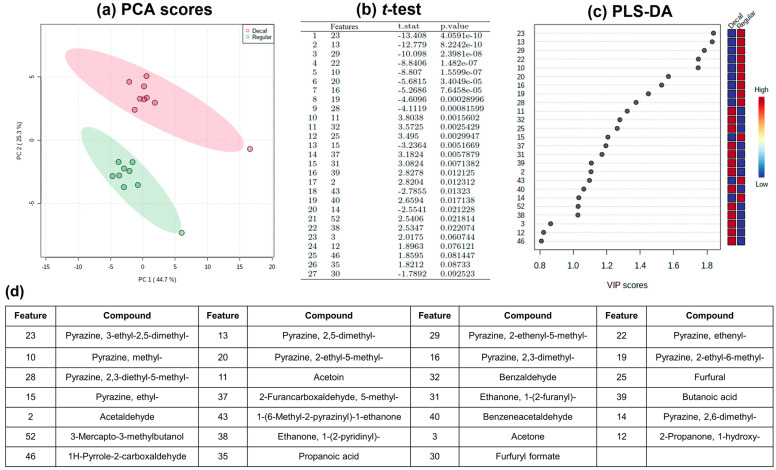
(**a**) PCA scores plot of the regular and the decaffeinated coffees using 52 aroma-related volatiles data set. (**b**) The selected features which significantly differed between the two coffee groups by performing the *t*-test and (**c**) PLS-DA. The numbers in the feature column of the *t*-test table and the *y*-axis of the PLS-DA VIP scores graph indicate the numbering of the aroma-related volatiles. (**d**) The volatile compounds corresponding to the significant features.

**Figure 3:**
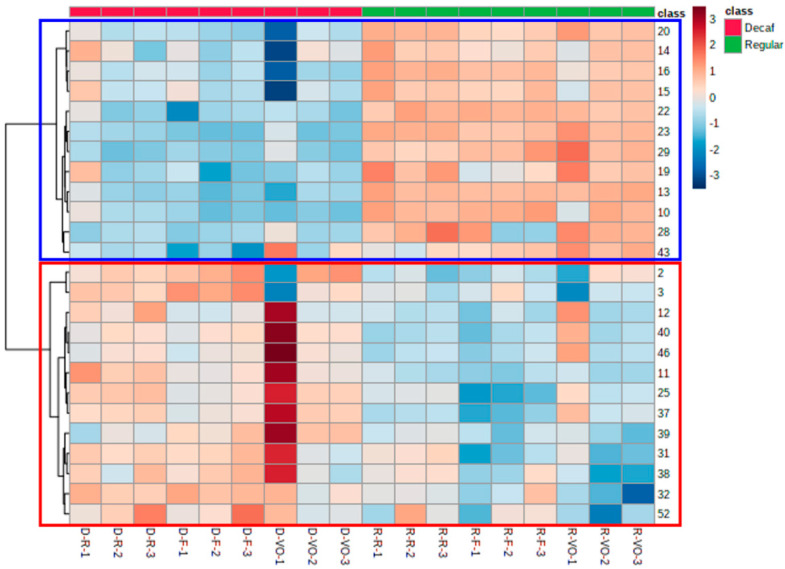
The heatmap of the top 25 significant aroma-related volatiles selected by feature selection tools. The *x*-axis indicates the samples names. The first letter of each sample name indicates the coffee type: regular (R) or decaffeinated (D) and the second letter corresponds to the label of the coffee capsule. The *y*-axis indicates the specific aroma-related volatiles (refer to Figure 2). The chemical structures of the volatiles are discussed in the text.

Using the data set of 52 aroma-related volatiles from three pairs of coffee (R-R vs. D-R, R-F vs. D-F, R-VO vs. D-VO), principal component analysis (PCA) screening was conducted to visualize a potential clustering trend between the regular and the decaffeinated coffee groups. As can be seen from the PCA scores plot, a significant clustering trend was observed between the two groups (Figure 2), which indicated that the aroma-related volatile contents were able to differentiate the two types of coffee. Applying the feature selection tools, a threshold of *p* < 0.1 was set for the univariate analysis, *t*-test, and a threshold of variable importance in projection (VIP) score > 0.8 was set for the multivariate analysis, partial least squares-discriminant analysis (PLS-DA). Both approaches presented the same top 25 significant features among the 52 aroma-related volatiles (Figure 2b–d). The heatmap of the top 25 features separated the volatiles into two clusters (Figure 3). The volatiles in the upper group highlighted in blue correspond to pyrazines. Their content was higher in the regular coffees and lower in the decaffeinated coffees. The lower group featured in red outlined several chemical families, such as aldehydes, ketones, furans, pyridines, acids, pyrroles, and sulfur-containing compounds. This group showed relatively higher content in the decaffeinated coffees compared to the regular coffees. 

#### 2.1.4. Predicted Aroma Difference between Regular and Decaffeinated Coffee

**Figure 4:**
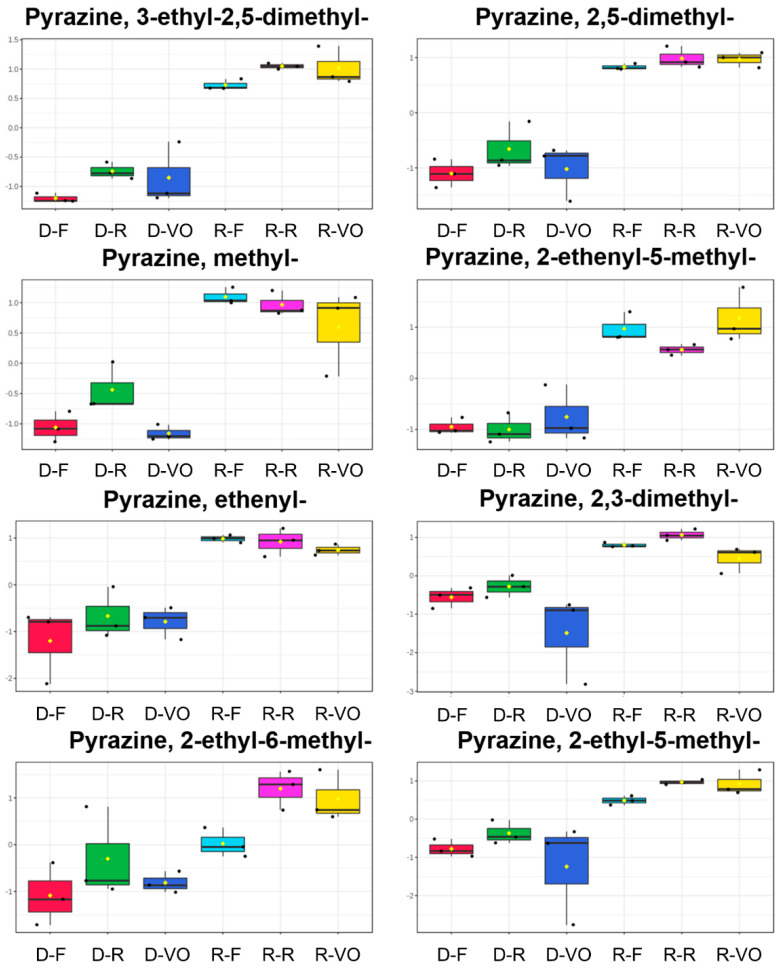
The comparison of the normalized contents of 8 pyrazines which presented a significant difference between R-R vs. D-R, R-F vs. D-F, and R-VO vs. D-VO. The first letter of each sample name indicates the coffee type: regular (R) or decaffeinated (D) and the second letter corresponds to the label of the coffee capsule.

**Figure 5:**
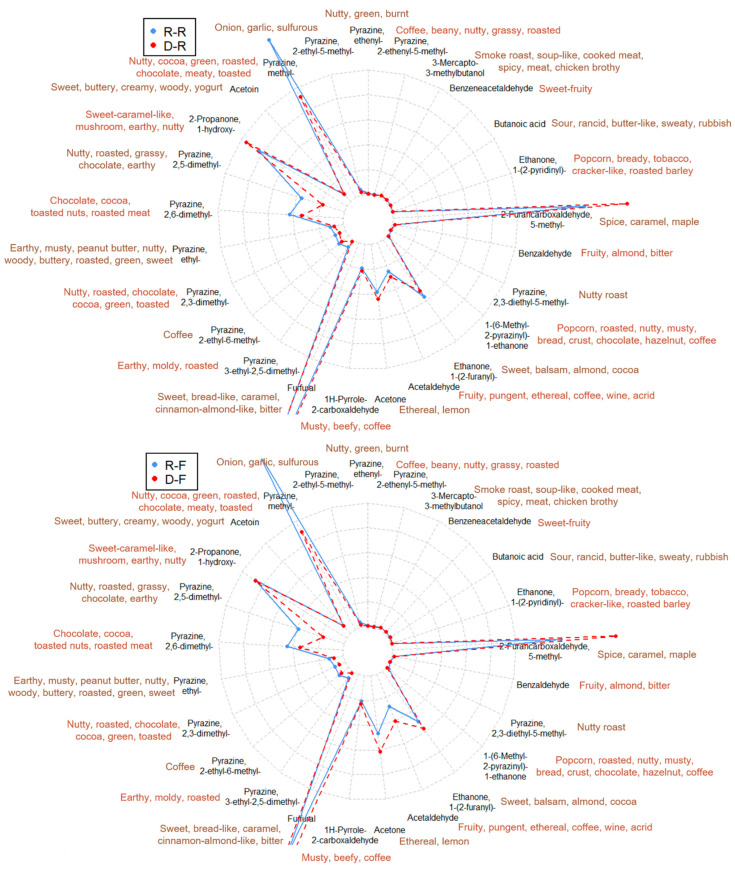

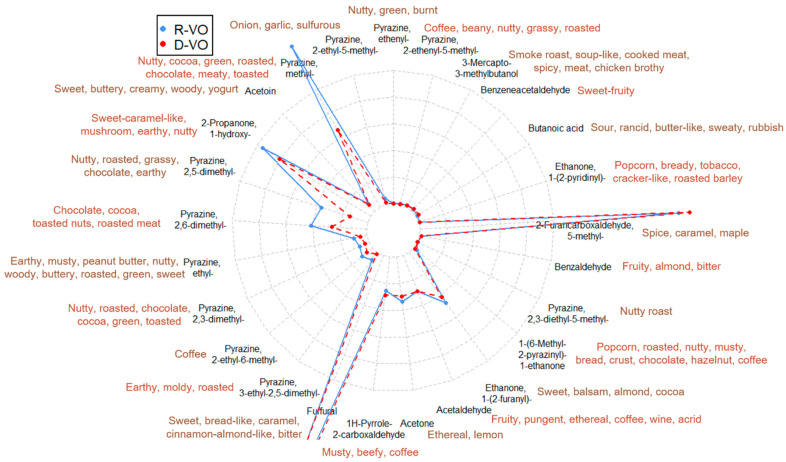
The radar graphs of coffees R-R vs. D-R, R-F vs. D-F, R-VO vs. D-VO based on the peak intensities of top 25 aroma-related volatiles. The aroma descriptions are obtained from references [8,20,21]. The first letter of each sample name indicates the coffee type: regular (R) or decaffeinated (D) and the second letter corresponds to the label of the coffee capsule.

As discussed previously, the reduced content of pyrazines was caused by the significantly reduced content of sucrose during the decaffeination process. Overall, 8 pyrazines out of 13 in the targeted aroma-related volatile list (Table 1) were found significantly different between R-R and D-R, between R-F and D-F, and between R-VO and D-VO, by one-way analysis of variance (ANOVA) with a threshold of *p* < 0.05 (Figure 4). They were identified by the NIST library spectral similarity of 700/1000 and linear retention indices (LRIs) ± 20. The 8 pyrazines are pyrazine, 3-ethyl-2,5-dimethyl-; pyrazine, 2,5-dimethyl-; pyrazine, methyl-; pyrazine, 2-ethenyl-5-methyl-; pyrazine, ethenyl-; pyrazine, 2,3-dimethyl-; pyrazine, 2-ethyl-6-methyl-; and pyrazine, 2-ethyl-5-methyl-. These pyrazines contribute to the nutty, roasted, chocolate, earthy, and musty aroma of the coffee [26,27,28,29] (Table 1). Therefore, in this aspect, the decaffeinated coffees D-R, D-F, and D-VO would exhibit a relatively thinner aroma. (Figure 5).

The amount of 2-furancarboxyaldehyde, 5-methyl- was higher in the decaffeinated coffee than in the regular coffee in all three pairs (Figure 5). This difference added more spice, caramel, and maple aroma in the decaffeinated coffee. Acetone and acetaldehyde were observed to be more concentrated in D-R and D-F than R-R and R-F. The fruity, ethereal, and wine aroma should be presented more intensively in the decaffeinated coffee.

### 2.2. Non-Targeted Analysis of Two Coffee Groups

#### 2.2.1. Classification Model and Feature Selection

To account for the instrumental variability, all sample triplicates were considered for the implementation of the classification model, leading therefore to a total of *n =* 48 observations and 630 features. The model was generated using the TreeBagger function in Matlab that implements the RF classification algorithm [30]. RF was chosen because it performs well for high-dimensional datasets, where the number of features outweighs the number of observations [31,32]. Its ability to overcome the dimensionality issue is attributed to the fact that only a subset of the features are considered to build each decision tree.

**Figure 6:**
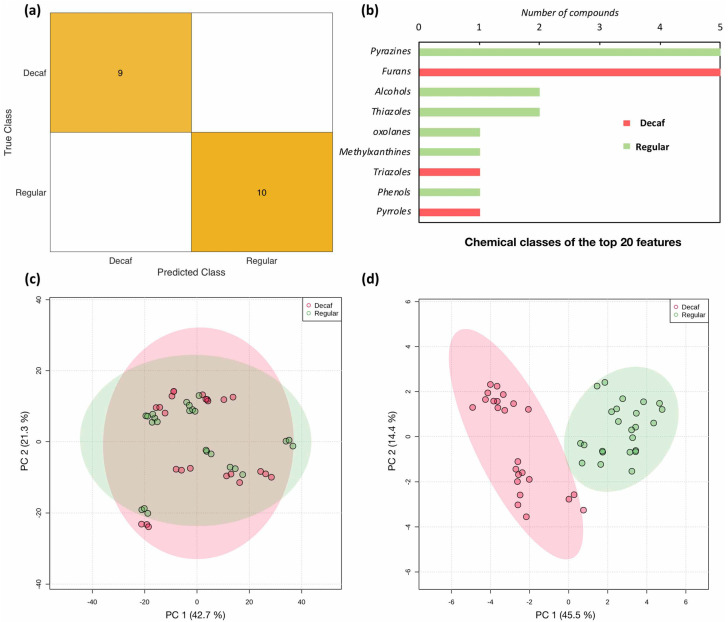
(**a**) The confusion matrix of the validation set (*n* = 19) obtained using the random forest classification algorithm. (**b**) The chemical classes of the top 20 discriminatory features. In red: the compounds that have the highest peak areas in decaffeinated coffee samples. In green: the compounds that have the highest peak areas in regular coffee samples. (**c**) PCA score plot using 630 features. (**d**) PCA score plot using only the top 20 discriminatory features.

The misclassification probability of the trained classifier is computed using the out-of-bag error (oobError). Owing to the calculation of the oobError, it can be considered that the TreeBagger algorithm has a built-in support for cross-validation, hence its implementation does not require splitting the data into training and validation sets. The classifier oobError was equal to 0, suggesting that all samples were accurately classified. Even though the TreeBagger algorithm has comparable functionalities to cross-validation, to endorse the accuracy of the obtained classifier, the dataset was randomly partitioned into a 60% training set (*n =* 29) and a 40% validation set (*n =* 19). In this scheme, the oobError of the training set was equal to 0.0690 and the classification model exhibited very high accuracy, as shown in the corresponding confusion matrix (Figure 6a).

In order to select the features that best divide the data, predictor importance is estimated based on the impact that the permutation of out-of-bag predictor observations has on the Mean Squared Error (MSE). In other words, the values for each individual feature are permuted across all the observations in the selected dataset. After the permutation, the MSE is computed. The larger this value, the more important the feature. The OOBPermutedVarDeltaError Matlab function was used to assess feature importance. It calculates the MSE averaged over all trees in the classification ensemble and divides it by the standard deviation (SD) taken over the trees, for each variable. 

A cutoff of 0.35 was set to select the 20 most discriminatory features (Figure 6b). These features belonged to different chemical families and included five pyrazines, five furans, two alcohols, two thiazoles, one oxolane, one methylxanthine, one triazole, one phenol and one pyrrole. The features names and numbers are listed in the Supplementary Materials Section (Table S1). Some of these chemical families were already investigated in the targeted analysis section (Section 2.1). Nonetheless, a major presence of pyrazine- and furan-derived compounds was observed among these features. Additionally, the efficiency of the selected features in accurately dividing the complete data set into two distinct groups (regular and decaf) was confirmed using PCA score plots (Figure 6c,d). It is worth noting that, based on Figure 6d, the presence of three subgroups in the decaffeinated coffee samples was suspected. Their presence was confirmed by performing a PCA only on the decaffeinated coffee samples, as shown in Figure S1 in the Supplementary Materials Section. The main difference between these groups consisted of the used decaffeination method. In fact, all the samples in group I belong to the same vendor, and caffeine was removed using the same method (LiCO_2_/water). Samples in group III are instrumental triplicates of the same sample, and scCO_2_ was used for the decaffeination process. Samples in group II were subjected to decaffeination methods that are different from group I and III.

As highlighted by the targeted analysis, the regular coffee samples presented higher peak areas in alcohols, pyrazines and phenols. Their higher presence was previously discussed in Section 2.1. Similar to pyrazines, thiazoles significantly contribute to the aroma profile of coffee. Their presence is attributed to the Strecker degradation [33]. Additionally, they are strongly related to the roasting intensity of coffee [34]. The presence of an oxolane-derived compound was also noticed among the selected top features with higher peak areas in the regular coffee samples. In fact, oxolanes originate from the thermal degradation of melanoidins due to the roasting process and are therefore linked to the roasting intensity of the coffee beans [35]. As previously discussed, decaffeinated coffee is more likely to be less roasted than regular coffee, thus this could explain the higher presence of thiazoles and oxolanes in regular coffee samples. Caffeine was also present among the top 20 features. Its peak areas were evidently higher in regular coffee samples. 

On the other hand, decaffeinated coffee samples displayed a more prominent presence of furan and pyrrole derivatives, corroborating therefore the conclusions drawn by the targeted analysis. Furans are one of the most distinguishable volatile aroma components in coffee. Their presence is related to the thermal degradation of endogenous components during the roasting process [36]. Based on the selected discriminatory features, furan derivatives seem to be a strong marker of the decaffeinated group. 

For the selected decaffeinated coffees, the manufacturers report their products with the flavor profiles of sweet aroma of almonds, chocolate and dried fruits for the D-L sample, cacao notes for the D-F sample, notes of caramel, chocolate and toasted bread for the D-I sample, and fruity notes for the D-R, D-VO and D-VI samples. Some of these flavors were not reported in their regular coffee counterparts. As for the other samples, i.e., D-DE and D-S, allergens such as peanuts and sesame were reported in the allergen section of the products. Little information regarding the impact of the coffee type on furan presence is available in the literature. However, it has been proven that furans are characteristic flavor markers for roasted almonds [37], roasted sesame seeds [38], chocolate [39] and are also responsible for the fruity aroma [40]. Therefore, this might be a conceivable explanation for their presence in the selected decaffeinated coffee samples. Additionally, furans are associated with a broad range of aroma profiles, ranging from caramel-like, to sweet and fruity, to nutty and meaty. Their prominent presence can also be interpreted as a way to compensate for the thin taste of decaffeinated coffees caused by the removal of caffeine and other aroma precursors during the decaffeination process. Nevertheless, the authors believe that a larger number of decaffeinated coffee samples covering a wider range of manufactures and a wider flavor and aroma spectra should be investigated in order to draw more reliable conclusions. The reason higher presence of a triazole derivative compound in the decaffeinated coffee samples is unclear to the authors. Some sorts of triazoles are used as fungicides in coffee farms in few regions of the world [41]. From all the studied regular and decaffeinated coffee samples, only D-L (Arabica and Robusta) and R-L (Arabica) samples presented higher levels of the triazole derivative. This difference might be related to the differences in farm management of specific coffee regions.

In order to explore whether a linear relationship exists between the top 20 features, Pearson correlation coefficients were calculated. The correlation matrix is provided in the Supplementary Materials Section (Figure S2). Based on the calculated coefficients, negative correlations ranging between r = −0.67 and r = −0.80 between pyrazine- and furan-derived compounds were depicted. These values suggest a strong correlation between these two chemical families. In other words, higher presence of furans entails a lower presence of pyrazines and vice versa. These results further corroborate the targeted analysis conclusions.

#### 2.2.2. Prediction Model

The RF algorithm was also used to build a prediction model that aimed at predicting the coffee type (regular or decaf) of 10 selected samples. This model was built using the original 16 samples and 630 features data set investigated in the present work. Once again, all sample triplicates were considered for the implementation of the model (*n =* 48). The complete data set was divided into a six-sample subset (*n =* 18) used to train the model and a 10-sample subset (*n =* 30) used to validate the model. 

Three pairs of coffee, namely (R-R and D-R), (R-DE and D-DE), and (R-L and D-L) were selected to train the model. These samples were carefully selected to account for the two types of coffee beans: Arabica and Arabica Robusta, and the three different decaffeination processes: LiCO_2_/water, DCM/water and scCO_2_ (Table 2). The misclassification error of the training set was equal to 0. The validation set consisted of five pairs of coffee, namely (R-F and D-F), (R-VO and D-VO), (R-VI and D-VI), (R-S and D-S) and (R-I and D-I). Its misclassification error was also equal to 0. The prediction model was able to accurately predict the type of all the coffee samples in the validation set. The ROC curve of the validation set is presented in the Supplementary Materials Section (Figure S3). 

The workflow built with this prediction model can be further applied to a wider range of decaffeinated coffees subjected to different decaffeination methods. In fact, using water-based or CO_2_-based decaffeination is considered to be healthier for coffee drinkers compared to solvent (DCM)-based methods. In this regard and for future applications, this model can help distinguish between the different decaffeination methods, and hence contribute to ensuring food quality and detecting potential food fraud.

## 3. Materials and Methods

### 3.1. Chemicals and Samples

The *n*-alkanes mixture (C_7-30_, 1000 μg mL^−1^ in hexane, Millipore Sigma, Bellefonte, PA, USA) was diluted to 100 μg mL^−1^ in hexane for the calculation of linear retention indices (LRIs). Sixteen coffee samples packed in coffee capsules were produced by five different vendors and purchased from retail supermarkets. The coffee samples represented eight pairs of regular (R) and decaffeinated (D) coffee (Table 2). Three pairs (R-R vs. D-R, R-F vs. D-F, R-VO vs. D-VO) were used to investigate the difference in aroma-related compounds between regular and decaffeinated coffee. In each pair of the above samples, the origin of the coffee beans, the species of the coffee beans (*Coffea arabica* L. (Arabica) or *Coffea canephora Pierre* (Robusta)), and the roasting process were identical. The only difference was whether they were processed with or without decaffeination process. All eight pairs of samples were used to differentiate between regular and decaffeination groups. All the samples were measured in triplicate.

### 3.2. HS-SPME-GC×GC-TOFMS Instrumentation

Right after opening the coffee capsules, the ground coffee powder was transferred into septum-sealed headspace vials. Each vial (20 mL) contained 1.0000 ± 0.0100 g of ground coffee. Samples were firstly incubated at 50 °C for 40 min with an agitator speed of 250 rpm. Then, the headspace of each sample was extracted by a 50/30 μm DVB/CAR/PDMS SPME fiber (Supelco, Bellefonte, PA, USA) at the same condition for 60 min before injection into GC. The new SPME fiber was conditioned according to the supplier’s instructions prior to use. During measurement, the fiber was pre-conditioned and post-conditioned at 250 °C for 10 min.

A Pegasus 4D GC×GC-TOFMS system with flow modulator (LECO Corp., St. Joseph, MI, USA) was employed in this study. In order to achieve an adequate separation of the studied samples, multiple column sets were investigated. However, the final set consisted of a reversed-phase (polar × non-polar) column set composed of first dimension (^1^D) StabilWax MS (30 m, 0.25 mm i.d., 0.25 μm df, Restek Corp., Bellefonte, PA, USA) and second dimension (^2^D) Rxi-5Sil MS (1.3 m, 0.1 mm i.d., 0.1 μm df, Restek Corp.) Two columns were installed in two separated ovens. The coffee headspace sample was desorbed from SPME fiber in the GC injector at 250 °C for 10 min. The main GC oven was set at 50 °C for 1 min, then increased to 230 °C with a ramp of 2 °C min^−1^, and finally increased to 250 °C (held for 5 min) at a ramp of 50 °C min^−1^. The secondary oven temperature offset was 5 °C. The transfer line temperature was 250 °C. The flow modulation period (P_M_) was 2 s with an injection duration of 0.08 s. The temperature of 70 eV electron ionization (EI) source was 250 °C. Mass range was 30–550 mu. MS acquisition rate was 150 spectra s^−1^ (Table S2). The 2D chromatogram of coffee sample R-R is presented in Figure S4.

### 3.3. Data Processing, Chemometrics, and Machine Learning

The data were processed using ChromaTOF^®^ (ver. 5.51, LECO Corp.). The putative identification of targeted aroma-related compounds was conducted by spectral similarity library search on NIST17 and LRIs confirmation. The chromatogram alignment and non-targeted peak table of all the coffee samples were generated on ChromaTOF Tile (ver. 1.01, LECO Corp.).

The chemometric tools including *t*-test, one-way ANOVA, PCA, PLS-DA, HCA, and heat map were operated using R version 4.0.2 (R Foundation for Statistical Computing, Vienna, Austria) and MetaboAnalyst 5.0 (Xia Lab, McGill University, Montréal, QC, Canada). Data pre-processing of normalization to sample median, log10 transformation, and autoscaling were conducted prior to applying chemometric tools. The distance measure and clustering algorithm used in the heatmap were Euclidean and Ward. D, respectively.

The final data matrix for the non-targeted analysis included a total of 16 samples (Table 2) and 630 features. Prior to statistical analyses, the compounds’ peak areas were normalized using Probabilistic Quotient Normalization (PQN) [42], log10 transformation, and autoscaling. The data were processed using ChromaTOF^®^ Tile (ver. 1.01.00.0, LECO Corp.). 

In order to assess feature importance, the RF machine-learning algorithm was used to select the most discriminatory features that would help distinguish between regular and decaffeinated coffee samples. It was also used to build a classification model that enabled the prediction of the coffee type (decaf or regular) of a subset of chosen coffee samples. Briefly, RF is a classification algorithm that consists of implementing a large number of decorrelated classification trees, also called decision trees, operating as an ensemble. These trees are generated by using randomly selected subsets of features and observations. Each individual decision tree in the RF accounts for a class prediction. The class selected by the majority of the trees will express the model’s prediction [30]. RF was implemented in an in-house Matlab (ver. R2019b, MathWorks, Natick, MA, USA) code using the TreeBagger function. Additionally, feature importance was assessed using the OOBPermutedVarDeltaError function.

## 4. Conclusions

The state-of-the-art HS-SPME-GC×GC-TOFMS instrument has made it possible to obtain the fingerprint chromatogram of a natural product, such as coffee in this study. It further enabled both targeted and non-targeted analysis to distinguish the decaffeinated coffees from regular ones. Two coffee groups were separated by 52 key aroma-related volatiles. In particular, the pyrazines showed significantly reduced content in decaffeinated coffee due to the removal of sucrose together with caffeine during the decaffeination process. The reduction in pyrazines can explain the thin aroma of decaffeinated coffees due to the lack of nutty, roasted and chocolate notes. For the non-targeted analysis, the use of the random forest machine-learning algorithm enabled the selection of 20 discriminatory features, among which a major presence of pyrazine and furan derivatives were noticed. These features allowed for an accurate classification of the studied coffee samples. Additionally, pyrazines were identified to be a marker of the regular coffee group, whereas furans were depicted as a marker of the decaffeinated coffee group. 

The highlighted differences in the presence of certain volatile compounds in the two coffee types along with their potential impact on the coffee aroma profile can be of great interest for the coffee industry. Understanding why and how certain volatile compounds are less concentrated in decaffeinated coffee can help coffee vendors to improve the decaffeination process to shorten the gap between the aroma profile of decaffeinated coffee and regular coffee. A pilot study on conducting the decaffeination of the green coffee beans using the main methods, specifically water-based, solvent-based, and liquid/supercritical carbon dioxide techniques, will be interesting in the future. The decaffeination effect on roasted coffee aroma by different decaffeination techniques will also be worth investigating. The approaches applied in this study to distinguish decaffeinated coffee from regular coffee could also be applied to other types of natural products. However, it is worth mentioning that applying them to study more complex natural products or to establish multiple classifications could be more challenging.

## Figures and Tables

**Table 1:** The targeted aroma-related volatile compound list.

Chemical Family	Compound	Aroma Description	CAS
Acids	Acetic acid	Pungent, sour, acidic, vinegar	64-19-7
	Propanoic acid	Pungent, acidic, cheesy, vinegar, sour milk, butter-like	79-09-4
	Butanoic acid	Sour, rancid, butter-like, sweaty, rubbish	107-92-6
	Butanoic acid, 3-methyl-	Acidic, sweaty, rancid, cheese, herbaceous	503-74-2
Alcohols	2,3-Butanediol (isomer)	Fruity, creamy, buttery	513-85-9
	2,3-Butanediol (isomer)	Fruity, creamy, buttery	513-85-9
	2-Furanmethanol	Caramellic, burnt, smoky, sweet, coffee	98-00-0
Aldehydes	Acetaldehyde	Fruity, pungent, ethereal, coffee, wine, acrid	75-07-0
	Butanal, 2-methyl-	Malty, fermented, buttery-oily	96-17-3
	Butanal, 3-methyl-	Almond, fruity, buttery-oily, malty, pungent, acrid, apple-like, sweaty	590-86-3
	Benzaldehyde	Fruity, almond, bitter	100-52-7
	Benzeneacetaldehyde	Sweet-fruity	122-78-1
	Propanal, 2-methyl-	Grassy, fermented, buttery-oily	78-84-2
Furans	Furan, 2-methyl-	Pungent, fruity	534-22-5
	2-Furfurylthiol ^1^	Smoke roast, caramel, burned matter, fresh coffee	98-02-2
	Furfural	Sweet, bread-like, caramel, cinnamon-almond-like, bitter	98-01-1
	Furan, 2-[(methylthio)methyl]- ^1^	Smoke roast	1438-91-1
	Furfuryl formate	Floral	13493-97-5
	Ethanone, 1-(2-furanyl)-	Sweet, balsam, almond, cocoa	1192-62-7
	2-Furanmethanol, acetate	Ethereal-floral, herbal-spicy, green	623-17-6
	2-Furancarboxaldehyde, 5-methyl-	Spice, caramel, maple	620-02-0
	Furaneol	Sweet, caramel	3658-77-3
Ketones	Acetone	Ethereal, lemon	67-64-1
	2,3-Butanedione	Buttery-oily, fruity, caramel	431-03-8
	2,3-Pentanedione	Buttery-oily, caramel-like	600-14-6
	Acetoin	Sweet, buttery, creamy, woody, yogurt	513-86-0
	2-Propanone, 1-hydroxy-	Sweet-caramel-like, mushroom, earthy, nutty	116-09-6
	1-Hydroxy-2-butanone	Sweet, coffee, toasted	5077-67-8
Lactones	Butyrolactone	Caramel, fatty, creamy, oily, sweet	96-48-0
Phenols	Phenol, 2-methoxy-	Phenolic, spicy, burnt, smoky	8021-39-4
	Phenol, 4-ethyl-2-methoxy-	Phenolic, spicy, sweet	2785-89-9
	2-Methoxy-4-vinylphenol	Phenolic, clove, spicy	7786-61-0
Pyridines	Pyridine, 3-ethyl-	Rotten fish, smoky, leather, tobacco, caramel, burnt, coffee-like, toasted	536-78-7
	Ethanone, 1-(2-pyridinyl)-	Popcorn, bready, tobacco, cracker-like, roasted barley	1122-62-9
Pyrazines	Pyrazine, methyl-	Nutty, cocoa, green, roasted, chocolate, meaty, toasted	109-08-0
	Pyrazine, 2,5-dimethyl-	Nutty, roasted, grassy, chocolate, earthy	123-32-0
	Pyrazine, 2,6-dimethyl-	Chocolate, cocoa, toasted nuts, roasted meat	108-50-9
	Pyrazine, ethyl-	Earthy, musty, peanut butter, nutty, woody, buttery, roasted, green, sweet	13925-00-3
	Pyrazine, 2,3-dimethyl-	Nutty, roasted, chocolate, cocoa, green, toasted	5910-89-4
	Pyrazine, 2-ethyl-6-methyl-	Earthy, musty, mold, flowery, fruity, hazelnut-like, toasted	13925-03-6
	Pyrazine, 2-ethyl-5-methyl-	Onion, garlic, sulfurous	13360-64-0
	Pyrazine, ethenyl-	Nutty, green, burnt	4177-16-6
	Pyrazine, 3-ethyl-2,5-dimethyl-	Earthy, moldy, roasted	13360-65-1
	Pyrazine, 2-ethenyl-6-methyl-	Coffee	13925-09-2
	Pyrazine, 2,3-diethyl-5-methyl-	Nutty roast	18138-04-0
	Pyrazine, 2-ethenyl-5-methyl-	Coffee, beany, nutty, grassy, roasted	13925-08-1
	1-(6-Methyl-2-pyrazinyl)-ethanone	Popcorn, roasted, nutty, musty, bread, crust, chocolate, hazelnut, coffee	22047-26-3
Pyrroles	1H-Pyrrole, 1-methyl-	Smoky, woody, herbal, sweet, animal, coffee	96-54-8
	Ethanone, 1-(1H-pyrrol-2-yl)-	Nutty, bread, walnut, licorice, cracker, popcorn-like	1072-83-9
	1H-Pyrrole-2-carboxaldehyde	Musty, beefy, coffee	1003-29-8
Sulfur-containing compounds	Methanethiol	Freshness, sulfurous, fresh coffee	74-93-1
	3-Mercapto-3-methylbutanol	Smoke roast, soup-like, cooked meat, spicy, meat, chicken brothy	34300-94-2

^1^ It is also a sulfur-containing compound.

**Table 2:** Coffee sample list.

Group	Sample	Bean Species	Bean Origin	Decaffeination Process ^1^
Regular	R-R	Arabica	Latin America/India/Eastern Africa	-
Decaf	D-R	Arabica	Latin America/India/Eastern Africa	LiCO_2_/water ^2^
Regular	R-F	Arabica	Latin America	-
Decaf	D-F	Arabica	Latin America	LiCO_2_/water
Regular	R-VO	Arabica	Brazil/Colombia	-
Decaf	D-VO	Arabica	Brazil/Colombia	LiCO_2_/water
Regular	R-VI	Arabica	Ethiopia/Mexico	-
Decaf	D-VI	Arabica	Colombia/Ethiopia	LiCO_2_/water
Regular	R-S	Arabica		-
Decaf	D-S	Arabica		Unknown
Regular	R-DE	Arabica, Robusta		-
Decaf	D-DE	Arabica, Robusta		DCM/water ^3^
Regular	R-I	Arabica		-
Decaf	D-I	Arabica		LiCO_2_
Regular	R-L	Arabica		-
Decaf	D-L	Arabica, Robusta		scCO_2_

^1^ The decaffeination process mentioned by the vendors; ^2^ LiCO_2_/water indicate the vendor applied either LiCO_2_ or water-based decaffeination process; ^3^ DCM/water indicate the vendor applied either DCM or water-based decaffeination process.

## Data Availability

The data presented in this study are available on request from the corresponding author.

## Supplementary Materials

The following are available online at https://www.mdpi.com/article/10.3390/molecules27061806/s1, Table S1: The top 20 discriminatory features selected using the random forest algorithm. Table S2: The analytical condition of HS-SPME-GC×GC-TOFMS. Figure S1: PCA score plots of the decaffeinated coffee samples. Figure S2: The correlation matrix between the top 20 features. Figure S3: The ROC curve of the validation set (*n =* 30) generated by using the Random forest algorithm. Figure S4: The 2D chromatogram of coffee R-R.
