# Supplementary material for: Distinguishing between Decaffeinated and Regular Coffee by HS-SPME-GC×GC-TOFMS, Chemometrics, and Machine Learning
Source: Molecules. 2022 Mar 10;27(6):1806. doi: 10.3390/molecules27061806 (PMC8948847; doi:10.3390/molecules27061806)
Supplement: Supplementary file 1 [file molecules-27-01806-s001.zip › molecules-1612094-supplementary.pdf]

# Supplementary Materials

# Distinguishing between Decaffeinated and Regular Coffee by HS-SPME-GC×GC-TOFMS, Chemometrics, and Machine Learning

**Yun Zou [1,*,†], Meriem Gaida [1,†], Flavio A. Franchina [2], Pierre-Hugues Stefanuto [1] and Jean-François Focant [1]**

[1] Organic and Biological Analytical Chemistry Group, MolSys Research Unit, University of Liège, 4000 Liège, Belgium; meriem.gaida@uliege.be (M.G.); phstefanuto@uliege.be (P.-H.S.); jf.focant@uliege.be (J.-F.F.)
[2] Department of Chemical, Pharmaceutical and Agricultural Sciences, University of Ferrara, 44121 Ferrara, Italy; flavioantonio.franchina@unife.it
* Correspondence: y.zou@uliege.be or yun.zou.titech@gmail.com
† These authors contributed equally to this work.

## Contents

**Table S1.** The top 20 discriminatory features selected using the random forest algorithm.

**Table S2.** The analytical conditions of HS-SPME-GC×GC-TOFMS.

**Figure S1.** PCA score plots of the decaffeinated coffee samples.

**Figure S2.** The correlation matrix between the top 20 features.

**Figure S3.** The ROC curve of the validation set (*n=30*) generated by using the Random forest algorithm.

**Figure S4.** The 2D chromatogram of the coffee sample R-R.

**Table S1.** The top 20 discriminatory features selected using the random forest algorithm.

| Feature N° | Name | CAS N° | Similarity/reverse similarity/probability | Library | Aroma/flavor profile | Chemical family |
|---|---|---|---|---|---|---|
| 1 | 1-Dodecanol,3,7,11-trimethyl- | 6750-34-1 | 501/523/12.64 | Replib | - | Alcohols |
| 2 | 2-Acetyl-4-methylthiazole | 7533-07-5 | 684/890/59.23 | Wiley9 | Nutty | Thiazoles |
| 3 | 2-methyl-5H-6,7-dihydrocyclopentapyrazine | 23747-46-8 | 861/861/46.07 | Wiley9 | - | Pyrazines |
| 4 | Furfurylmethyldisulfide | 57500-00-2 | 852/852/45.55 | Wiley9 | Broth-like, aromatic, roasted, fatty | Furans |
| 5 | 4,4-Ethylenedioxy-1-pentylamine | 66442-97-5 | 568/647/25.18 | Mainlib | - | Oxolane[1] |
| 6 | 1-Propanol | 71-23-8 | 775/775/3.88 | Wiley9 | Alcohol, candy, pungent | Alcohols |
| 7 | Benzofuran,2,3-dihydro- | 496-16-2 | 686/686/8.53 | Wiley9 | - | Furans |
| 8 | Bis(2-furfuryl) disulfide | 4437-20-1 | 730/730/28.59 | Replib | Roasted meaty, coffee | Furans |
| 9 | Pyrazine,2-methyl-6-(1-propenyl)-, (E) | 18217-81-7 | 778/778/46.43 | Mainlib | - | Pyrazines |
| 10 | 5-Hydroxymethylfurfural | 67-47-0 | 729/729/28.02 | Replib | - | Furans |
| 11* | Caffeine | 58-08-2 | 801/801/93.17 | Wiley9 | Coffee | Methylxanthines |
| 12 | Thiazole,4-methyl | 693-95-8 | 822/822/60.31 | Wiley 9 | Green, nut, roasted meat | Thiazoles |
| 13 | Pyrazine,2-ethenyl-5-methyl | 13925-08-1 | 875/875/54.17 | Mainlib | Savory | Pyrazines |
| 14 | 5-methyl-5H-6,7-dihydrocyclo-pentapyrazine | 23747-48-0 | 898/898/27.07 | Wiley9 | Nuts, peanuts, roast | Pyrazines |
| 15* | Caffeine | 58-08-2 | 898/898/94.68 | Wiley9 | coffee | Methylxanthines |

*In the data processing by the ChromTile software the caffeine peak was cut in two tiles.
[1] It also contains a primary amine group, [2] it also contains a ketone group.

| | | | | | | |
|---|---|---|---|---|---|---|
| 16 | Pyrazine, trimethyl | 14667-55-1 | 938/938/98.18 | Mainlib | Coca, earth, must, potato, roast | Pyrazines |
| 17 | Ethanone,1-(2-furanyl)- | 1192-62-7 | 957/957/86.87 | Wiley9 | Balsamic, cocoa, coffee | Furans[2] |
| 18 | 1H-Benzotriazole,4-methyl- | 29878-31-7 | 859/859/30.42 | Wiley9 | - | Triazoles |
| 19 | 2-Methoxy-4-vinylphenol | 7786-61-0 | 887/887/61.69 | Mainlib | Clove, curry, spice | Phenols |
| 20 | 3-Acetyl-1-methylpyrrole | 932-62-7 | 892/892/52.01 | Mainlib | Woody | Pyrroles |

**Table S2.** The analytical conditions of HS-SPME-GC×GC-TOFMS.

| | |
|---|---|
| **HS-SPME condition** | |
| SPME fiber | 50/30 m DVB/CAR/PDMS |
| Pre-condition temperature | 250 ºC |
| Pre-condition time | 10 min |
| Sample incubation temperature | 50 ºC |
| Incubation time | 40 min |
| Agitator speed | 250 rpm |
| Agitator on time | 10 s |
| Agitator off time | 2 s |
| Fiber sample extraction time | 60 min |
| Sample desorb time | 10 min |
| Post-condition temperature | 250 ºC |
| Post-condition time | 10 min |
| **GC condition** | |
| Injector temperature | 250 ºC |
| Injector mode | Split |
| Split ratio | 5 :1 |
| Oven temperature ramp | 50 ºC (1 min, 2 ºC/min) 230 ºC (50 ºC/min) 250 ºC (5 min) |
| Flow control mode | Constant flow |
| Carrier gas flow rate | 1 mL/min |
| Column set | $^{1}$D : StabilWax MS (30 m, 0.25 mm i.d., 0.25 m $d_f$)<br>$^{2}$D : Rxi-5Sil MS (1.3 m, 0.1 mm i.d., 0.1 m df) |
| **MS condition** | |
| Transferline temperature | 250 ºC |
| Ion source temperature | 250 ºC |
| Electron energy | 70 eV |
| Emission current | 1 mA |
| Mass range | *m/z* 30 - 500 |
| Acquisition time | 3 min 20 s – 96 min 24 s |
| Solvent delay | 3 min 20 s |
| Acquisition rate | 150 spectra/s |
| Extraction frequency | 32 kHz |
| **Modulator condition** | |
| Modulation period | 2 s |
| Injection duration | 0.08 s |

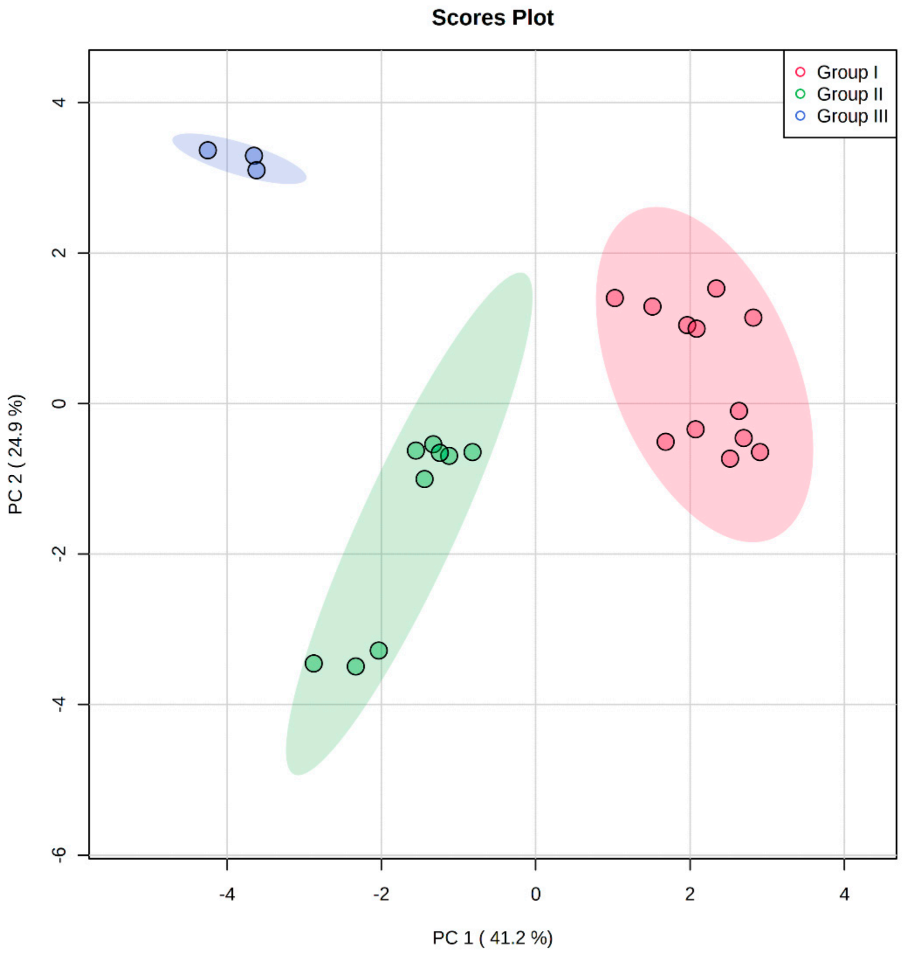


**Figure S1.** PCA score plots of the decaffeinated coffee samples.

Group I: D-VI, D-R, D-F and D-VO coffee samples.

Group II: D-I, D-DE and D-S coffee samples.

Group III: D-L coffee samples.

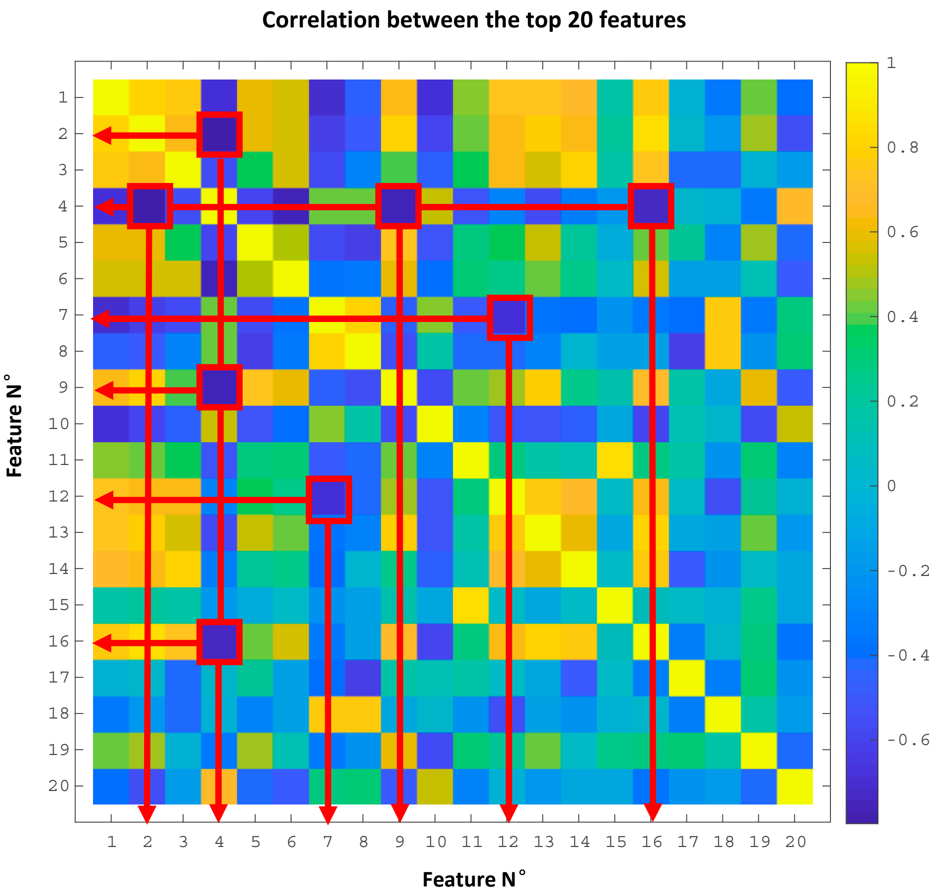


**Figure S2.** The correlation matrix between the top 20 features.

r = -0.72 between feature N°4 and N°16

r = -0.76 between feature N°4 and feature N°9

r = -0.80 between feature N°4 and feature N°2

r = -0.67 between feature N°7 and feature N°12

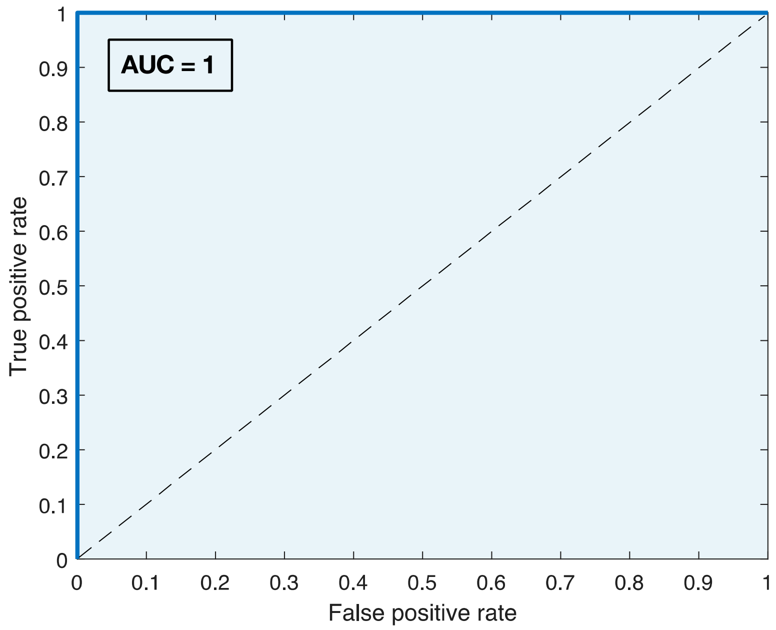


**Figure S3.** The ROC curve of the validation set (*n=30)* generated by using the Random forest algorithm.

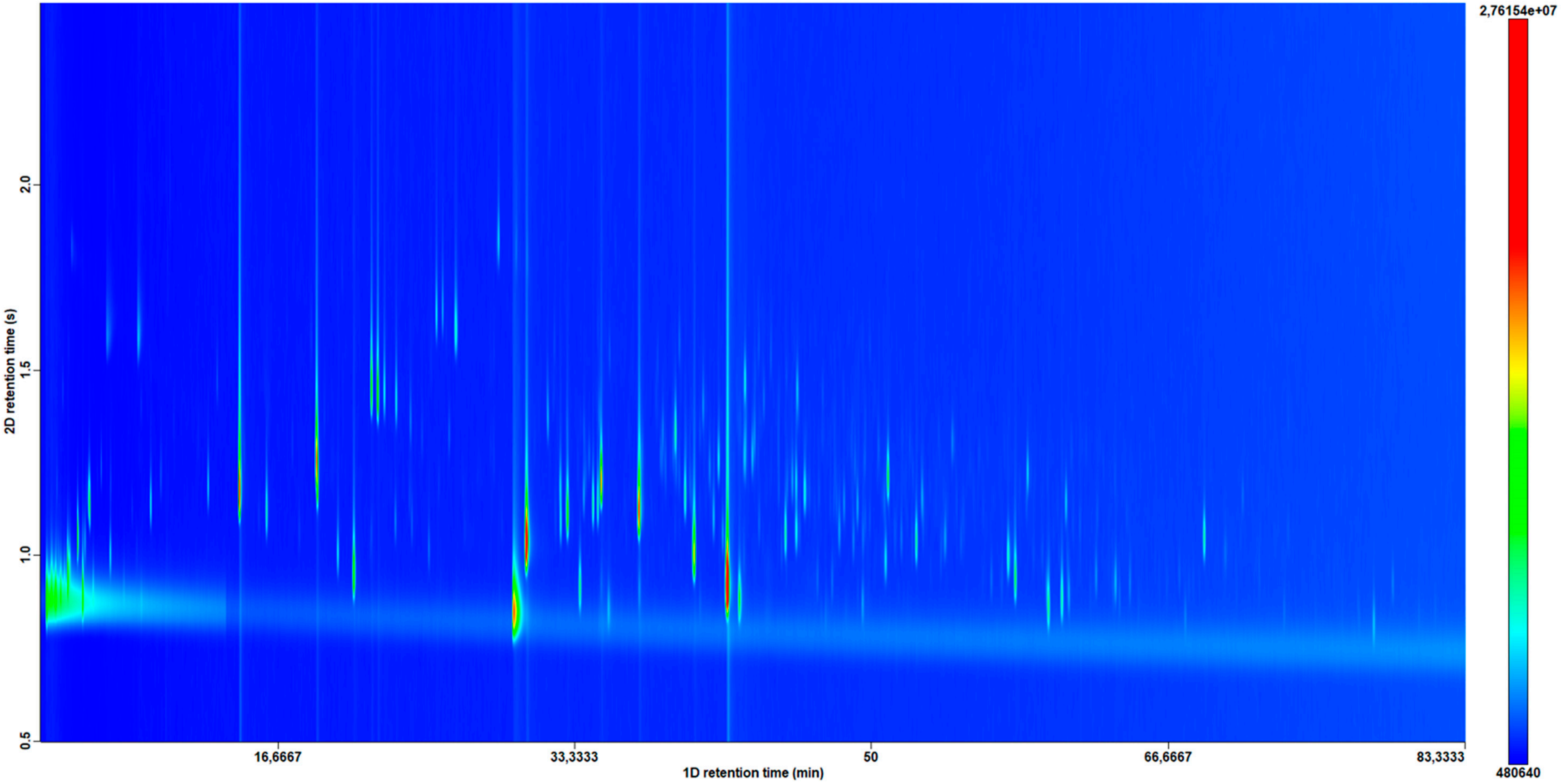


**Figure S4.** The 2D chromatogram of the coffee sample R-R.
